# Association of Divergent Carcinoembryonic Antigen Patterns and Lung Cancer Progression

**DOI:** 10.1038/s41598-020-59031-1

**Published:** 2020-02-07

**Authors:** Yen-Shou Kuo, Ming-Yi Zheng, Mo-Fan Huang, Chia-Cheng Miao, Li-Hao Yang, Tsai-Wang Huang, Yu-Ting Chou

**Affiliations:** 1 Division of Thoracic Surgery, Department of Surgery, Tri-Service General Hospital, National Defense Medical Center, Taipei, Taiwan, ROC; 2 Institute of Biotechnology, National Tsing Hua University, Hsinchu, Taiwan, ROC

**Keywords:** Prognostic markers, Non-small-cell lung cancer

## Abstract

Changes in expression patterns of serum carcinoembryonic antigen at initial diagnosis (CEA^In^) and disease progression (CEA^Pd^) in lung cancer patients under EGFR-tyrosine kinase inhibitors (TKI) treatment may reflect different tumor progression profiles. Of the 1736 lung cancer patients identified from the cancer registry group between 2011 to 2016, we selected 517 patients with advanced stage adenocarcinoma, data on EGFR mutation status and CEA^In^, among whom were 288 patients with data on CEA^Pd^, eligible for inclusion in the correlation analysis of clinical characteristics and survival. Multivariable analysis revealed that CEA^In^ expression was associated with poor progression-free survival in patients harboring mutant EGFR. Moreover, CEA^In^ and CEA^Pd^ were associated with the good and poor post-progression survival, respectively, in the EGFR-mutant group. Cell line experiments revealed that CEA expression and cancer dissemination can be affected by EGFR-TKI selection. EGFR-mutant patients, exhibiting high CEA^In^ (≥5 ng/mL) and low CEA^Pd^ (<5 ng/mL), showed a potential toward displaying new metastasis. Taken together, these findings support the conclusion that EGFR mutation status is a critical factor in determining prognostic potential of CEA^In^ and CEA^Pd^ in patients under EGFR-TKI treatment, and CEA^In^ and CEA^Pd^ are associated with distinct cancer progression profiles.

## Introduction

Carcinoembryonic antigen (CEA) is one of the most commonly used tumor markers whose overexpression was first discovered in colorectal cancer and followed by cancers in the aerodigestive tract and other organs^1,2^. Serum CEA is used as a marker along with imaging for monitoring tumor progression and predicting survival outcomes in colorectal cancer^3,4^. Although the suitability of CEA as an effective marker for lung cancer remains debatable, characteristics such as lower cost, noninvasiveness, and lack of other reliable markers, make CEA an attractive option in lung cancer^5,6^. Additionally, CEA measurements before and after surgery have been suggested to provide valuable information for predicting disease progression and for determining the need for adjuvant treatment during early stage lung cancer^7,8^.

As proven by several large-scale clinical trials, epidermal growth factor receptor (EGFR)-tyrosine kinase inhibitors (TKI) are highly effective in lung cancer patients harboring EGFR activating mutations, mainly, deletions in exon 19 (Del19) and a L858R mutation in exon 21^9–13^. However, despite an initial positive response, patients harboring the activating EGFR mutations ultimately develop resistance to EGFR-TKI by different mechanisms^14–18^. Thus, the utility of CEA as a predictive or prognostic marker of EGFR-TKI treatment response remains controversial. Several studies have reported that serum CEA levels are closely associated with EGFR-TKI treatment outcomes, and that high CEA expression after EGFR-TKI treatment predicts poor survival in patients with EGFR-mutant lung cancer^19,20^. In contrast, some reports have shown that CEA levels do not respond to tumor progression during EGFR-TKI treatment^21,22^. Moreover, EGFR-TKI treatment may affect the clonality of heterogeneous lung tumors, thus modulating CEA expression^23^.

Therefore, this study aimed to further our understanding of CEA expression in different pathological subtypes in advanced lung cancer patients and to verify the effects of EGFR mutation status on the prognostic potential of CEA in these patients. Additionally, CEA levels at initial diagnosis (CEA^In^) and at disease progression (CEA^Pd^) were extracted to study the association between CEA heterogeneity, metastasis and remaining survival after disease progression in these patients. Finally, we used EGFR-mutant lung adenocarcinoma cells to monitor the change of CEA heterogeneity after EGFR-TKI treatment and its association with dissemination and chemoresistant properties of lung cancer cells.

## Results

### Patient selection and clinicopathological features

**Figure 1:**
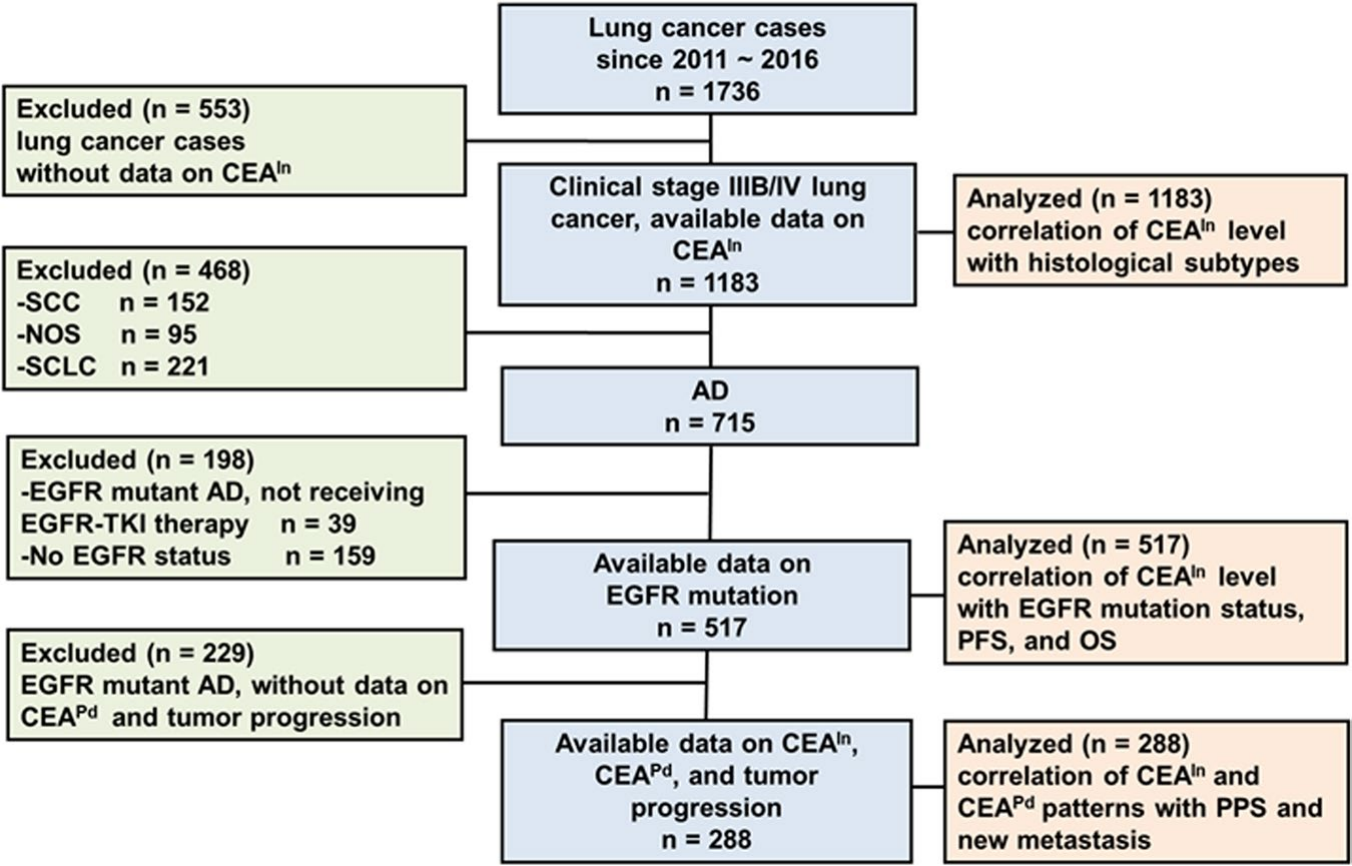
Flow chart of patient selection for analysis. Of the 1736 lung cancer patients from the cancer registry group, 517 patients with advanced stage adenocarcinoma had information on CEA^In^ (CEA level at initial diagnosis) and EGFR mutation status, but only 288 (55.7%, 288/517) patients with information on both CEA^In^ and CEA^Pd^ (CEA level at disease progression) were included in correlation analysis of clinical characteristics with survival outcome. CEA^In^, serum carcinoembryonic antigen level at initial diagnosis; CEA^Pd^, serum carcinoembryonic antigen level at disease progression; AD, adenocarcinoma of lung, SQ, squamous cell carcinoma of lung; NOS: non-small cell lung cancer-not otherwise specified; SCLC, small cell lung cancer; PFS, progression-free survival; OS, overall survival; PPS, post-progression survival.

Of the 1736 lung cancer patients from the cancer registry group at our institute, serum CEA information at initial diagnosis (CEA^In^) was available for 1183 patients with stage IIIB-IV cancer; of these, adenocarcinoma was diagnosed in 715 patients (Fig. 1). Among the 715 patients, 517 cases had data on EGFR mutation status, and this cohort included 288 (55.7%, 288/517) cases with information on serum CEA levels at disease progression (CEA^Pd^). Patients with advanced stage adenocarcinoma were classified based on EGFR mutation status (n = 517) and CEA^Pd^ information (n = 288), and the demographic profiles of these patients, including age, gender, clinical stage, smoking, EGFR status, and CEA^In^ and CEA^Pd^ levels, are listed in Table 1. Of the 517 eligible patients, 190 (36.8%) harbored wild-type EGFR and 327 (63.2%) harbored EGFR mutations (Table 1); the latter group comprised 175 patents with an exon 19 in-frame deletion (Del19), and 152 with an exon-21 point-mutation (L858R). All enrolled stage IIIB-IV patients harboring EGFR mutations received EGFR-TKI (the first or second generation) treatment as first-line care during the study period. Patients with wild-type EGFR and advanced disease received cytotoxic chemotherapy.

**Table 1:** Clinical characteristics of patients with advanced lung adenocarcinoma eligible for further analysis.

Variable	With available data on EGFR mutation (n = 517)	With available data on CEA^Pd^ (n = 288)
Age (years)*	66 [59, 77]	64.5 [58, 73.8]
**Age**		
<65 years	231 (44.7%)	144 (50.0%)
≥65 years	286 (55.3%)	144 (50.0%)
**Sex**		
Female	275 (53.2%)	165 (57.3%)
Male	242 (46.8%)	123 (42.7%)
**Stage**		
IIIB	84 (16.2%)	47 (16.3%)
IV	433 (83.8%)	241 (83.7%)
**Smoking**		
Never	336 (65.0%)	198 (68.8%)
Ever	181 (35.0%)	90 (31.3%)
**EGFR**		
Wild-type	190 (36.8%)	94 (32.6%)
Mutant	327 (63.2%)	194 (67.4%)
**CEA**^**In**^		
<5 ng/mL	199 (38.5%)	96 (33.3%)
5–100 ng/mL	215 (41.6%)	134 (46.5%)
>100 ng/mL	103 (19.9%)	58 (20.1%)
**CEA**^**Pd**^		
<5 ng/mL	NA	92 (31.9%)
5–100 ng/mL	NA	126 (43.8%)
>100 ng/mL	NA	70 (24.3%)
Follow-up Months*	15.4 [8.3, 25.5]	20.8 [13, 32.8]

EGFR, epidermal growth factor receptor; CEA^In^, serum CEA level at initial diagnosis; CEA^Pd^, serum CEA level at disease progression; NA, not applicable.

^*^Data were presented as median [25^th^ and 75^th^ percentiles].

### CEA^In^ as a prognostic marker in adenocarcinoma patients harboring EGFR-mutant

To investigate the possible association between CEA^In^ expression and specific histological subtypes, relevant data from 1183 patients in the lung cancer registry group were subjected to comparison analysis in the various histological subtypes. The results demonstrated that adenocarcinoma was the dominant subtype with a significantly higher CEA^In^ level than other types (Supplementary Fig. 1A). A similar analysis of CEA^In^ expression and EGFR mutation status in adenocarcinoma patients (n = 517) revealed that serum CEA^In^ levels were higher in the EGFR-mutant than in the wild-type group (Supplementary Fig. 1B).

To evaluate the effect of EGFR mutation status on the prognostic potential of CEA^In^, adenocarcinoma patients were stratified into either EGFR wild-type (n = 190) or EGFR-mutant (n = 327) groups and subjected to multivariable Cox regression analysis. We found that abnormal CEA^In^ expression was associated with poor PFS only in the EGFR-mutant (CEA^In^ > 100 ng/mL, adjusted hazard ratio [aHR] = 2.39, 95% confidence interval [CI], 1.65–3.45, *P* < 0.0071) but not in the EGFR wild-type group (Table 2). Similarly, in EGFR wild-type patients, CEA^In^ expression did not correlate with OS, whereas in patients harboring EGFR mutants, CEA^In^ served as an independent factor that could predict poor prognosis (CEA^In^ > 100 ng/mL, aHR = 1.90, 95% CI, 1.29–2.78, *P* < 0.0071) (Table 2).

**Table 2:** Association between clinical characteristics and progression-free survival or overall survival stratified by EGFR mutation status among 517 patients with advanced stage lung adenocarcinoma.

Predictor	Progression-free survival				Overall survival			
	EGFR wild-type (n = 190)		EGFR mutant (n = 327)		EGFR wild-type (n = 190)		EGFR mutant (n = 327)	
	aHR (95% CI)	*P* value	aHR (95% CI)	*P* value	aHR (95% CI)	*P* value	aHR (95% CI)	*P* value
Age ≥ 65 years	1.30 (0.90–1.88)	0.165	1.33 (1.01–1.73)	0.039	1.43 (1.03–2.00)	0.035	1.45 (1.08–1.93)	0.012
Male sex	1.65 (1.05–2.59)	0.031	1.25 (0.89–1.76)	0.202	1.36 (0.90–2.05)	0.143	1.37 (0.97–1.95)	0.076
Stage IV	1.96 (1.22–3.17)	0.006*	1.22 (0.82–1.82)	0.325	2.45 (1.54–3.90)	<0.001*	3.49 (1.83–6.64)	<0.001*
Ever smoking	0.83 (0.53–1.30)	0.405	0.95 (0.66–1.38)	0.798	1.23 (0.82–1.83)	0.315	0.98 (0.67–1.42)	0.906
CEA^In^								
<5 ng/mL	Reference		Reference		Reference		Reference	
5–100 ng/mL	1.08 (0.72–1.62)	0.717	1.37 (1.01–1.87)	0.045	1.04 (0.73–1.50)	0.814	1.34 (0.95–1.88)	0.094
>100 ng/mL	1.05 (0.57–1.93)	0.879	2.39 (1.65–3.45)	<0.001*	1.34 (0.82–2.19)	0.243	1.90 (1.29–2.78)	0.001*

HR, hazard ratio; aHR, adjusted hazard ratio; CI, confidence interval; EGFR, epidermal growth factor receptor; CEA^In^, serum carcinoembryonic antigen level at initial diagnosis;

^*^Statistically significant after a Bonferroni adjustment at *P* < 0.0071.

**Figure 2:**
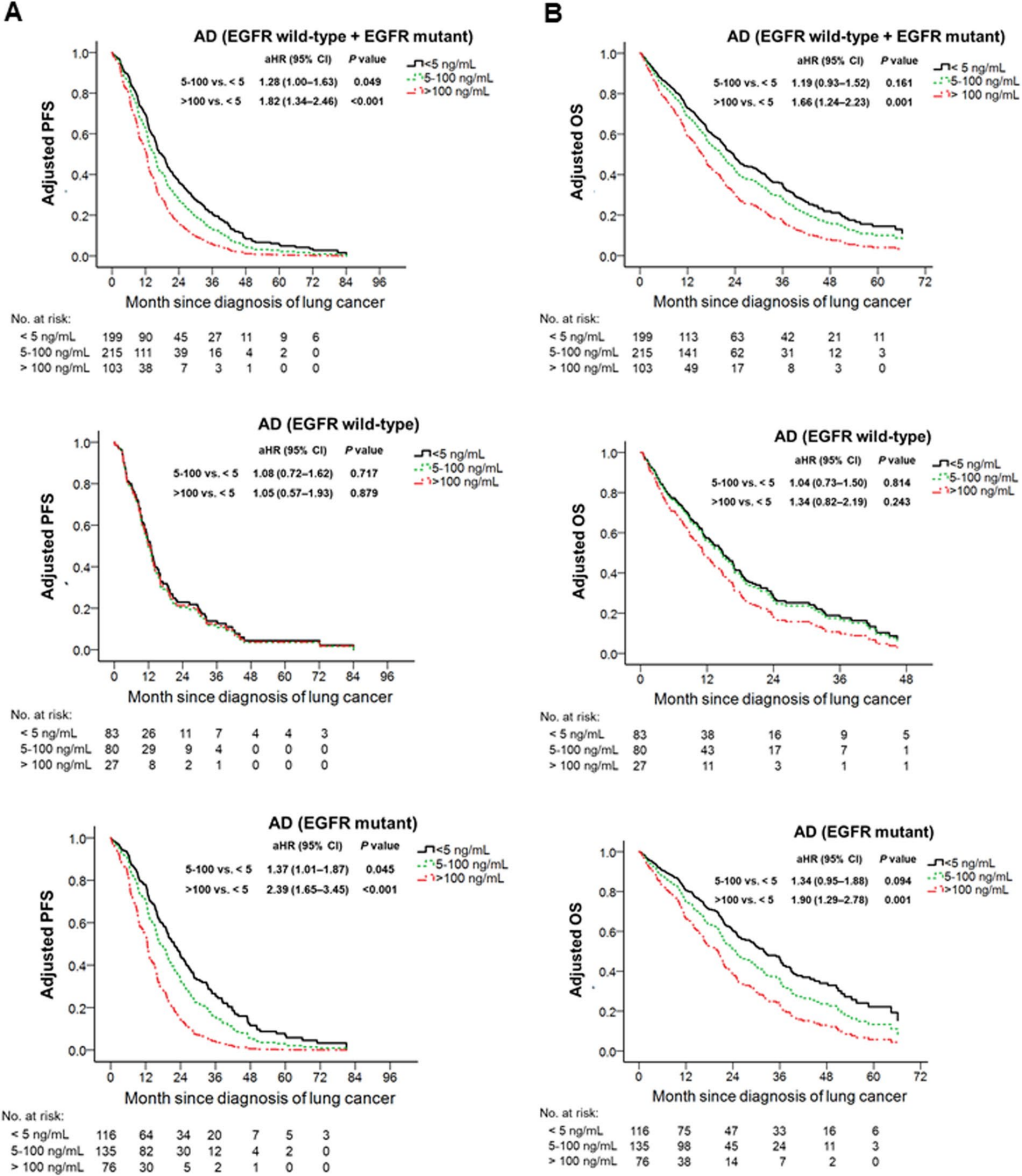
Effect of EGFR mutation status on prognostic value of CEA^In^ in patients with advanced stage lung adenocarcinoma. (**A**) Adjusted progression-free survival (PFS) was derived from multivariable Cox model to assess the correlation between CEA^In^ expression and PFS in all lung adinocarcinoma patients (top left), EGFR wild-type patients (middle left), and EGFR mutants (bottom left). (**B**) Adjusted overall survival (OS) derived from multivariable Cox model to assess correlation between CEA^In^ expression and OS in all lung adenocarcinoma patients (top right), EGFR wild-type patients (middle right), and EGFR mutants (bottom right). Variables included in the model were age, sex, stage, smoking and CEA^In^ expression. The statistical significance was set at *P* < 0.0071.

Adjusted survival analysis derived from the multivariable Cox model revealed that high CEA^In^ expression correlated with poor PFS and OS in adenocarcinoma patients (Fig. 2A,B, top), attesting to the potential of CEA^In^ as a prognostic factor in adenocarcinoma patients. However, when patients were further stratified into EGFR wild-type or mutant groups, the prognostic value of CEA^In^ for PFS and OS was lost in the EGFR wild-type group but remained in the EGFR-mutant group (Fig. 2A,B, middle and bottom). In EGFR-mutant group, the mean values (standard deviation) of CEA^In^ in good, moderate and poor histologic grades were 12.6 (28.4), 9 (31.4), and 19.8 (30.4) ng/mL, respectively, with a *P*-value of 0.786, suggesting no association of CEA^In^ with histologic grades. These findings indicate that EGFR mutation status is a critical factor in determining the prognostic potential of CEA^In^.

### Correlation of CEA expression with the post-progression survival

**Figure 3:**
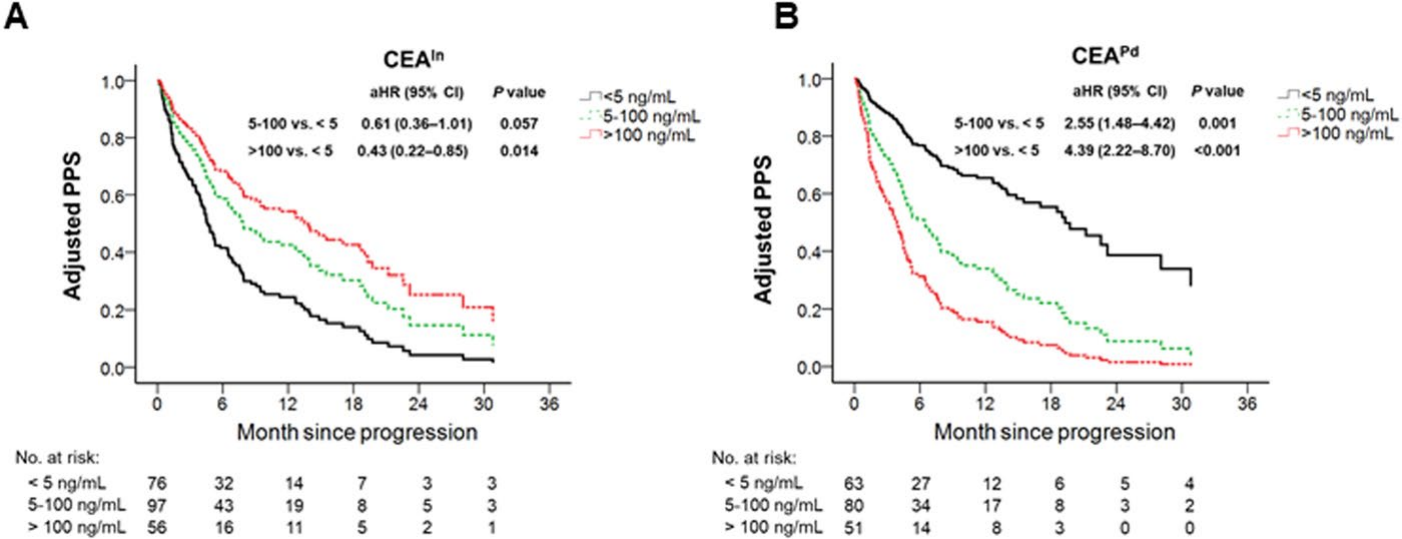
CEA levels at initial diagnosis and disease progression predict different post-progression survival. (**A**,**B**) Adjusted survival derived from multivariable Cox model to assess correlation of CEA^In^ (**A**) and CEA^Pd^ (**B**) expression with post-progression survival (PPS) in patients harboring EGFR mutations. Variables included in the model were age, sex, stage, smoking, CEA^In^ and CEA^Pd^ expression. The statistical significance was set at *P* < 0.0071.

As CEA expression can change during disease progression, we compared the prognostic value of both CEA levels, namely at initial diagnosis (CEA^In^) and at disease progression (CEA^Pd^), along with other clinicopathological factors, in predicting post-progression survival (PPS). To test whether EGFR mutation status affects the prognostic potential of CEA^In^ and CEA^Pd^ in predicting PPS, adenocarcinoma patients were stratified into EGFR wild-type (n = 94) and EGFR-mutant (n = 194) groups and subjected to multivariable Cox model. We observed that CEA^Pd^ levels (>100 ng/mL, aHR = 4.39, 95% CI, 2.22–8.70, *P* < 0.0071; 5–100 ng/mL, aHR = 2.55, 95% CI, 1.48–4.42, *P* < 0.0071) were significantly correlated with good and poor predicting post-progression survival (PPS), respectively, but only in the EGFR-mutant group and not in the wild-type group (Table 3). Further, adjusted survival analysis derived from the multivariable Cox model showed that high CEA^Pd^ expression correlated with poor PPS in patients harboring EGFR mutations, whereas high CEA^In^ expression was associated with good PPS in patients harboring EGFR mutations (Fig. 3A,B). Moreover, we observed that different EGFR-TKI treatments (gefitinib, erlotinib, or afatinib) did not have significant differences on PFS and PPS (Supplementary Table 1). These findings indicate that CEA^Pd^ level would be an effective prognostic marker of PPS, especially in adenocarcinoma patients harboring EGFR mutations. Moreover, the observation that CEA^Pd^ and CEA^In^ levels predict different PPS suggests that EGFR-TKI treatment leads to changes in CEA expression patterns.

**Table 3:** Association between clinical characteristics and post-progression survival among 288 patients with advanced stage lung adenocarcinoma and available data of CEA^In^ and CEA^Pd^, stratified by EGFR mutation status.

Predictor	EGFR wild-type (*n* = 94)		EGFR mutant (*n* = 194)	
	aHR (95% CI)	*P* value	aHR (95% CI)	*P* value
Age ≥ 65 years	1.82 (1.04–3.19)	0.036	0.82 (0.57–1.18)	0.277
Male sex	1.73 (0.95–3.15)	0.072	1.32 (0.83–2.11)	0.241
Stage IV	2.39 (1.19–4.81)	0.015	3.43 (1.63–7.22)	0.001*
Ever smoking	1.17 (0.63–2.16)	0.624	0.68 (0.41–1.14)	0.142
CEA^In^				
<5 ng/mL	Reference		Reference	
5–100 ng/mL	1.50 (0.70–3.23)	0.300	0.61 (0.36–1.01)	0.057
>100 ng/mL	1.13 (0.39–3.25)	0.823	0.43 (0.22–0.85)	0.014
CEA^Pd^				
<5 ng/mL	Reference		Reference	
5–100 ng/mL	1.28 (0.59–2.79)	0.531	2.55 (1.48–4.42)	0.001*
>100 ng/mL	1.94 (0.75–5.03)	0.175	4.39 (2.22–8.70)	<0.001*

HR, hazard ratio; aHR, adjusted hazard ratio; CI, confidence interval; EGFR, epidermal growth factor receptor; CEA^In^, serum carcinoembryonic antigen level at initial diagnosis; CEA^Pd^, serum carcinoembryonic antigen level at disease progression;

^*^Statistically significant after a Bonferroni adjustment at *P* < 0.0071.

### EGFR-TKI treatment affects CEA expression and dissemination property of lung cancer cells

**Figure 4:**
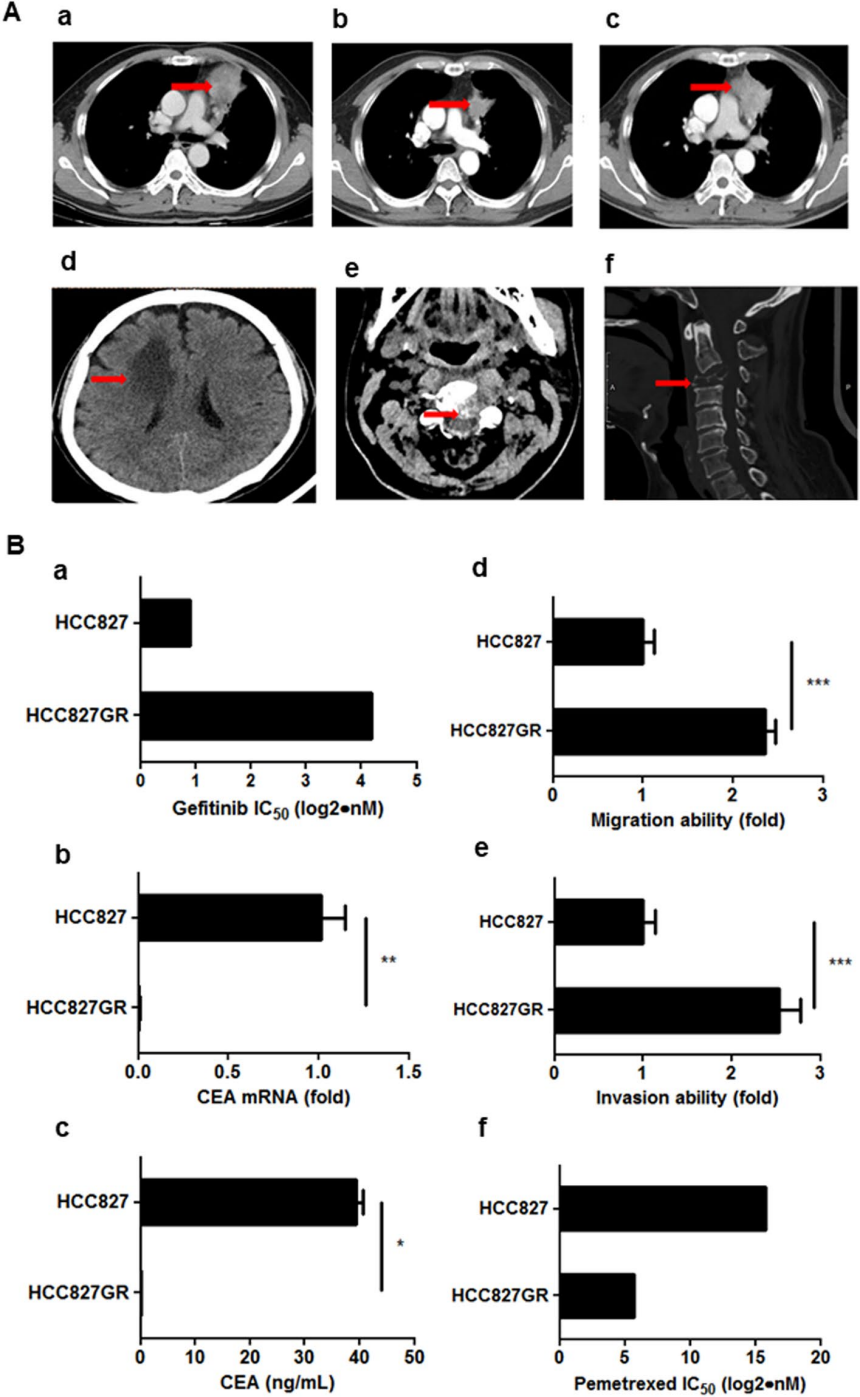
CEA heterogeneity during EGFR-TKI treatment. (**A**) (a) A 55-year-old male patient with clinical stage IV adenocarcinoma harbored EGFR-L858R mutation, and serum CEA level at initial diagnosis was 32.77 ng/mL. The red arrow indicates the primary tumor in the left upper lobe of the lung. (b) Four months of EGFR-TKI treatment with gefitinib led to decreased tumor size and normalization of the serum CEA level (2.99 ng/mL). (c–f) After eleven months of gefitinib treatment, the tumor progressed with an enlarged primary tumor and newly developed metastases in the brain and the C spine. However, serum CEA levels remained normal (1.94 ng/mL). The red arrow indicates tumors. (**B**) (a) IC_50_ analysis of gefitinib in HCC827 cells versus HCC827GR cells. (b) qPCR analysis to assess *CEA* mRNA expression in HCC827 cells versus HCC827GR cells. (c) ELISA analysis to assess CEA protein expression in HCC827 cells versus HCC827GR cells. (d) Transwell migration analysis to compare migration ability in HCC827 cells versus HCC827GR cells. (e) Boyden chamber invasion analysis to compare invasion ability in HCC827 cells versus HCC827GR cells. (f) IC_50_ analysis of pemetrexed in HCC827 cells versus HCC827GR cells.

The hypothesis that EGFR-TKI treatment can affect CEA expression was based on the case of a 55-year-old male, who was initially diagnosed with clinical stage IV adenocarcinoma. The primary tumor was found in the left upper lobe of the lung and metastatic lesions were seen in the bone (Fig. 4A). The patient harbored EGFR-L858R mutation, and serum CEA level at initial diagnosis was 32.77 ng/mL. Four months of EGFR-TKI treatment with gefitinib led to decreased tumor size and normalization of serum CEA levels. Seven months later, the tumor progressed with an enlarged primary tumor and newly developed metastases in the brain and the C spine. However, serum CEA levels remained normal. Therefore, to test whether EGFR-TKI treatment affects CEA expression pattern and dissemination ability of lung cancer cells harboring EGFR mutations, we treated EGFR-mutant HCC827 (delE746_A750) lung adenocarcinoma cells with increasing concentrations of gefitinib. Surviving cells were pooled together, propagated, and labeled HCC827GR. No EGFR-T790M mutation was detected in the HCC827GR cells (data not shown). We observed that while HCC827GR cells had a much higher IC_50_ for gefitinib, compared to the parental HCC827 cells, *CEA* expression was lost and migration and invasion abilities were increased in HCC827GR cells (Fig. 4B). The cell viability assay demonstrated that HCC827GR cells are more sensitive to pemetrexed than HCC827 cells (Fig. 4B). These data indicate that EGFR-TKI treatment can change CEA expression pattern and affect dissemination and chemoresistant properties of lung cancer cells.

### Correlation between CEA expression pattern and new metastasis during TKI treatment and disease progression

**Figure 5:**
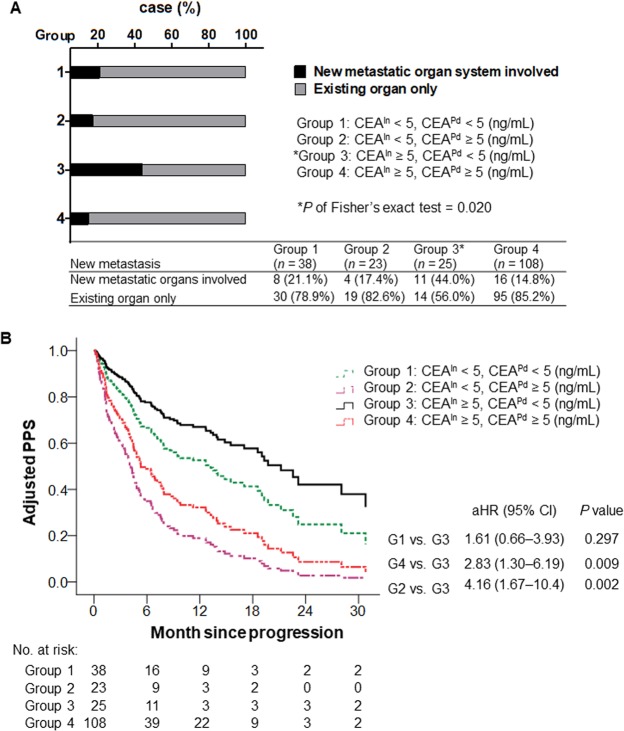
Association of CEA patterns with new metastasis and post-progression survival. (**A**) Correlation between CEA^In^ (< or ≥ 5 ng/mL), CEA^Pd^ (< or ≥ 5 ng/mL) levels and new metastasis in adenocarcinoma patients harboring EGFR mutations (n = 194). (**B**) Adjusted remaining survival after disease progression derived from multivariable Cox model to assess correlation between distinct CEA^In^ and CEA^Pd^ expression patterns from four groups with PPS in EGFR-mutant lung adenocarcinoma patients. Variables included in the model were age, sex, stage, smoking, CEA^In^ and CEA^Pd^ expression. The statistical significance was set at *P* < 0.0071.

To further confirm whether EGFR-TKI treatment affects CEA expression and metastasis in patients, we divided EGFR-mutant lung cancer patients into four groups based on their CEA^In^ (CEA^In^ < 5 ng/mL or CEA^In^ ≥ 5 ng/mL) and CEA^Pd^ levels (CEA^Pd^ < 5 ng/mL or CEA^Pd^ ≥ 5 ng/mL). We observed that patients with higher CEA^In^ (≥5 ng/mL) but normal CEA^Pd^ levels (<5 ng/mL) exhibited a greater trend toward development of new metastasis after EGFR-TKI treatment compared to other groups (Fig. 5A). Remaining survival analysis after disease progression in these four groups of patients revealed that patients in group 3 (CEA^In^ ≥ 5 ng/mL and CEA^Pd^ < 5 ng/mL) had a better PPS than other groups (Fig. 5B). These data support the notion that EGFR-TKI treatment affects CEA expression patterns and indicate that this change confers distinct survival and metastatic properties.

## Discussion

EGFR-TKI targeted therapy is one of the major treatment modalities in lung cancer. However, whether EGFR mutation status and EGFR-TKI treatment affect the prognostic potential of CEA, both before and after disease progression, remains less understood. Here, we demonstrate that CEA^In^ is a prognostic factor associated with poor PFS and OS in patients harboring EGFR mutations but not in those with wild-type EGFR. Further, CEA^In^ and CEA^Pd^ can serve as prognostic factors associated with good or poor PPS, respectively. We also show that, in lung cancer patients harboring EGFR mutations, the divergent CEA^In^ and CEA^Pd^ pattern (CEA^In^ ≥ 5 ng/mL and CEA^Pd^ < 5 ng/mL) is associated with new metastasis. Our results provide insights into CEA heterogeneity subsequent to EGFR-TKI treatment and may reflect oligoclonality of lung tumors with distinct progression patterns.

A previous immunohistochemistry analysis of early stage lung tumors revealed that CEA expression is higher in lung adenocarcinoma than squamous cell carcinoma^24^. We also show that adenocarcinoma patients exhibit higher serum CEA^In^ levels than squamous cell carcinoma patients or small cell lung cancer patients in the late stage. Additionally, the EGFR-mutant group had higher CEA^In^ levels than the wild-type group. Several reports have stated that CEA^In^ levels can serve as a prognostic factor associated with poor survival in NSCLC after chemotherapy or surgery, while others have indicated that CEA^In^ did not predict survival^25–31^. Cai reported that high CEA^In^ level (>5 ng/mL) was associated with EGFR mutation status and correlated with poor OS in stage I-IIIA NSCLC patients, who were not divided into EGFR-wild and mutant groups^32^. Zhao *et al*. found that high CEA^In^ level (>10 ng/mL) was associated with poor PFS and OS in stage IIIB/IV NSCLC patients with EGFR-mutations, whereas the association between CEA^In^ level with survival in the EGFR-wild group was not addressed^33^. Here, we report that higher CEA^In^ levels (CEA^In^ 5–100 ng/mL and CEA^In^ > 100 ng/mL) are associated with poor PFS and OS in stage IIIB/IV patients with adenocarcinoma. While stratifying adenocarcinoma patients into EGFR-mutant and wild-type groups, who were under EGFR-TKI and chemotherapies before diseases progression, respectively, we observed that CEA^In^ prognostic power was evident only in the EGFR-mutant group but not in the wild-type group. Our data reveal that higher CEA^In^ levels are associated with poor disease progression and three groups (CEA^In^ < 5 ng/mL, CEA^In^ 5–100 ng/mL, and CEA^In^ > 100 ng/mL) show distinct survival outcomes under EGFR-TKI treatment. Resistance toward first- and second-generation EGFR-TKI treatment can be attributed to EGFR-T790M-dependent and independent mechanisms^14–18^. Patients with non-EGFR-T790M tumors tend to develop new metastasis after EGFR-TKI treatment, implying the presence of differential oncogenic properties in EGFR-TKI-resistant lung cancer cells^34^.

As CEA levels may be affected by treatments for tumor progression, we also evaluated the prognostic value of CEA^Pd^ levels at the time of first disease progression and show that CEA^Pd^ is an independent factor associated with PPS. Moreover, when CEA^Pd^ is accounted for, CEA^In^ correlates with good PPS. Further, sub-stratification of adenocarcinoma into EGFR-mutant and wild-type groups revealed that the prognostic powers of CEA^In^ and CEA^Pd^ in predicting PPS were statistically significant in the EGFR-mutant group but not in the wild-type group. These observations imply that frequent assessments of CEA level may serve as an effective prognostic tool in adenocarcinoma patients with EGFR mutations. To the best of our knowledge this is the first report that shows distinct roles for initial baseline CEA in predicting survival before and after disease progression.

Whether CEA can serve as an effective marker for monitoring TKI treatment in EGFR-mutant lung adenocarcinoma is questionable because CEA heterogeneity may lead to contradictory results from different studies. By selecting EGFR-TKI resistant lung cancer cells *in vitro*, we could monitor changes in CEA heterogeneity in CEA-positive lung adenocarcinoma cells during gefitinib treatment. Using cell line models, we found that clones resistant to EGFR-TKI treatment lost CEA expression, and demonstrated increased migration and decreased pemetrexed chemoresistant properties. These data point to the presence of CEA heterogeneity and suggest that it can be affected by EGFR-TKI treatment. It has been reported that EGFR-TKI treatment can select CEA-positive clones from a CEA-negative primary tumor, resulting in tumor progression^23^. To further understand the role of CEA heterogeneity in lung tumors, we divided adenocarcinomas with EGFR mutations into four groups based on CEA^In^ and CEA^Pd^ levels and discovered that patients displaying high CEA^In^ but low CEA^Pd^ levels tended to develop new metastasis. Further, patients with low CEA^In^ but high CEA^Pd^ tended to have worse PPS. Many enrolled patients with disease progression after TKI treatment received pemetrexed chemotherapy. These findings support the notion that CEA expression patterns in patients can be altered by TKI treatment, and patients with changes in expression patterns of CEA^In^ and CEA^Pd^ may exhibit different responses toward subsequent chemotherapies after the EGFR-TKI treatment. We have previously reported that tumor heterogeneity is in part regulated by epigenetic control to generate lung cancer cells with distinct dissemination and chemoresistant properties^35^. Whether heterogeneous CEA expression patterns and related oncogenic properties in lung cancer cells are under epigenetic control requires further investigation.

Although we demonstrate a significant prognostic potential for CEA, there are limitations to our study, such as its single-institution retrospective design. Next, data were extracted from the lung cancer registry group of the Tri-Service General Hospital. Although patients were treated in accordance with national guidelines for lung cancer, they were treated by different physicians and departments, which may have led to different clinical management and outcomes. Besides, CEA^Pd^ data were not available for 229 of 517 patients with known EGFR status, and thus the results might be skewed. Although multivariable analysis revealed that CEA^Pd^ is independently associated with PPS in EGFR-mutant but not in wild-type group, the high aHR (1.92) of CEA^Pd^ (>100 ng/mL) in the wild-type group could be due to the low patient numbers. Moreover, records on T790M status in our study cohort were incomplete, and thus the changes in expression patterns of CEA^In^ and CEA^Pd^ cannot be correlated with T790M-dependent and -independent resistance mechanisms. Further, as whole-body imaging was not routinely performed in every patient, new metastatic sites may have been missed. We did not control for other factors that can lead to a false-positive increase in CEA, such as smoking, infections, inflammatory bowel disease, pancreatitis, cirrhosis of the liver, and some benign tumors in the organs in which an elevated CEA level indicates cancer.

To summarize, our findings indicate that CEA expression is associated with EGFR mutation status, and that its prognostic potential is limited to patients harboring EGFR mutations. Additionally, CEA heterogeneity can be affected by EGFR-TKI treatment. Changes in CEA expression patterns between inital diagnosis and disease progression may reflect oligoclonality of lung cancer cells with distinct chemoresistant and metastatic properties conferred by EGFR-TKI treatment.

## Materials and Methods

### Patients and samples

Records of patients (N = 1736) with lung cancer, registered at the cancer registry group, Tri-Service General Hospital, National Defense Medical Center, between January 2011 and January 2016, were screened. Patients were excluded if they had more than one primary cancer, no data on EGFR mutation or CEA levels, or if they did not undergo EGFR-TKI treatment while harboring an EGFR mutation. Patients with histologically or cytologically confirmed stage IIIB-IV adenocarcinoma of the lung were eligible for inclusion and data were collected from 517 patients by retrospective chart review, with a data cutoff limit of September 2018 for outcome. This retrospective study was conducted with the approval of the Joint Institutional Review Board of the Tri-Service General Hospital (Approved number 1-107-05-146). The need for informed consent was waived, and all methods were performed in accordance with the relevant guidelines and regulations. Serum CEA (normal range: 0–5 ng/mL) was routinely measured via an electrochemiluminescence immunoassay on an automatic analyzer (CEA-RIACT Cisbio Bioassays, France) at the time of diagnosis of lung cancer and disease progression according to the manufacture instruction. EGFR mutation analysis on tissue biopsy samples was performed at diagnosis using the ROCHE cobas EGFR Mutation Test kit according to the manufacture instruction. Patients with detectable EGFR exon 19–21 mutations were placed in the EGFR-mutant group. Patients with no detectable EGFR mutations were categorized as the EGFR wild-type group. Chest computed tomography was performed every three months in patients to monitor the cancer progression according to the policy of National Health Insurance. All selected patients with stage IIIB-IV disease and mutated-EGFR received EGFR-TKI (gefitinib, erlotinib, or afatinib) as first-line treatment during the study period. Most of them received platinum plus pemetrexed therapy after developing resistance to EGFR-TKI.

### Cell line assays

HCC827 cells were initially obtained from the American Type Culture Collection and later certified via STR-PCR DNA profiling and EGFR sequencing analysis. HCC827GR cells were established in our laboratory by exposing HCC827 cells to stepwise and incremental concentrations of gefitinib^18^. Surviving cells were pooled, propagated, and labeled HCC827GR. Cell culture, IC_50_ analysis, qPCR, migration and invasion assays were performed as described previously^18^. CEA ELISA was performed using human CEA ELISA kit (General Biologicals, Taiwan) according to the manufacture instruction.

### Statistical analysis

The difference in CEA^In^ levels among lung cancer subtypes or EGFR mutations was analyzed by the Kruskal–Wallis test followed by Bonferroni-adjusted multiple comparison or the Mann–Whitney U test, as needed. The associations between CEA, EGFR mutation status, progression-free survival (PFS), overall survival (OS), and post-progression survival (PPS) were determined using Cox regression analysis. Correlation analyses between CEA^In^, CEA^Pd^, and new metastasis in patients with advanced stage lung adenocarcinoma were performed using Fisher’s exact test. Progression-free survival (PFS) is defined as the time from the first EGFR-TKI administration to the date of first radiological confirmed progression. The post-progression survival (PPS) is defined as the time from the end of the last EGFR-TKI administration to the date of death. Overall survival (OS) is defined as the time from the first EGFR-TKI administration to the to the date of death.

There were a total of seven multiple testing results regarding the OS, PFS or PPS (Tables 2–3 and Fig. 5); therefore, a Bonferroni adjustment of type I error was done and a two-sided *P* value lower than 0.0071 (0.05/7) was considered statistically significant. Data analyses were conducted using SPSS ver.22 (Armonk, NY: IBM Corp).

## Supplementary information

Supplementary Information.
