# Supplementary material for: Association of Divergent Carcinoembryonic Antigen Patterns and Lung Cancer Progression
Source: Sci Rep. 2020 Feb 7;10:2066. doi: 10.1038/s41598-020-59031-1 (PMC7005848; doi:10.1038/s41598-020-59031-1)
Supplement: Supplementary file 1 — Supplementary Information. [file 41598_2020_59031_MOESM1_ESM.pdf]

Supplementary Information for

Association of Divergent Carcinoembryonic Antigen Patterns and Lung Cancer Progression

Yen-Shou Kuo, Ming-Yi Zheng, Mo-Fan Huang, Chia-Cheng Miao, Li-Hao Yang, Tsai-Wang Huang, Yu-Ting Chou

This file includes: Supplementary Table 1 and Supplementary Figure 1

Supplementary Table 1. Association between TKI therapy and progression-free survival or post-progression survival among 327 patients with EGFR mutation status

	Progression-free survival		Post-progression survival	
Predictor	aHR (95% CI)	*P* value	aHR (95% CI)	*P* value
Age ≥ 65 years	1.36 (1.03–1.80)	0.029	0.88 (0.63–1.24)	0.472
Male sex	1.16 (0.82–1.64)	0.404	1.27 (0.85–1.88)	0.241
Stage IV	1.41 (0.95–2.08)	0.089	3.31 (1.68–6.54)	0.001
Ever smoking	1.00 (0.69–1.46)	0.996	1.02 (0.67–1.56)	0.920
TKI therapy				
Gefitinib	Reference		Reference	
Erlotinib	1.24 (0.88–1.74)	0.214	0.76 (0.49–1.19)	0.227
Afatinib	1.26 (0.79–2.00)	0.333	0.61 (0.29–1.26)	0.179

TKI, tyrosine kinase inhibitors; aHR, adjusted hazard ratio; CI, confidence interval; EGFR, epidermal growth factor receptor;

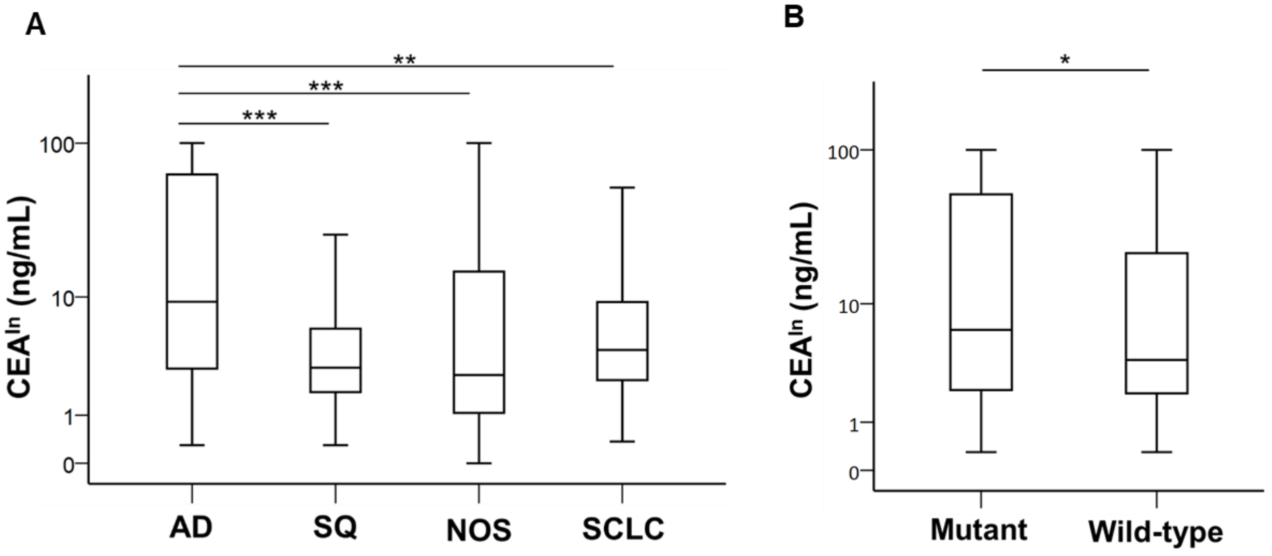

Supplementary Figure 1. Higher CEA^{ln} expression in patients harboring EGFR mutations **A**. Correlation analysis of CEA^{ln} expression with pathological subtypes of lung tumors (n = 1183) was performed using the Kruskal–Wallis test for pairwise comparison with the P value adjusted via the Bonferroni-method. CEA^{ln}, carcinoembryonic antigen level in serum at initial diagnosis; NSCLC, non-small cell lung cancer; AD, adenocarcinoma of lung, SQ, squamous cell carcinoma of lung; NSCLC-NOS: non-small cell lung cancer-not otherwise specified; SCLC, small cell lung cancer. **B**. Correlation analysis of CEA^{ln} expression with EGFR mutation status in adenocarcinomas with known EGFR status (n = 517) using the Mann–Whitney U test.
